# Assessment of HPV 16, HPV 18, p16 expression in advanced stage laryngeal cancer patients and prognostic significance^☆^^☆☆^

**DOI:** 10.1016/j.bjorl.2019.11.005

**Published:** 2019-12-12

**Authors:** Selman Dogantemur, Suleyman Ozdemir, Aysun Uguz, Ozgur Surmelioglu, Muhammed Dagkiran, Ozgur Tarkan, Ulku Tuncer

**Affiliations:** a Kadirli State Hospital, Department of Otorhinolaryngology Head & Neck Surgery, Osmaniye, Turkey; b Cukurova University School of Medicine, Department of Otorhinolaryngology Head & Neck Surgery, Adana, Turkey; c Cukurova University School of Medicine, Department of Pathology, Adana, Turkey

**Keywords:** Larynx, Squamous cell carcinoma, HPV, p16, Alcohol, Laringe, Carcinoma espinocelular, HPV, p16, Álcool

## Abstract

**Introduction:**

Human papilloma virus is an etiological risk factor for a subset of head and neck squamous cell carcinomas. HPV has been proven to be a powerful prognostic biomarker for oropharyngeal cancer, but its role in the larynx has not been explored in depth. The developmental mechanisms of laryngeal carcinomas are quite complex and controlled by various factors. Smoking and alcohol are most important risk factors. Recent studies indicate that HPV infection also plays an important role in larynx carcinomas. HPV related laryngeal carcinomas especially occur at the supraglottic region of larynx.

**Objective:**

We aimed to determine the frequency of HPV/protein16 positivity in patients with laryngeal carcinoma and association of HPV and/or p16 positivity with variables such as age, sex, smoking habits, tumor localization, lymph node metastasis, recurrence and survival in advanced stage laryngeal carcinoma in our study.

**Methods:**

This retrospective study included 90 patients with advanced laryngeal carcinoma. The Control group was 10 normal larynx mucosa specimens. The presence of HPV was investigated polyclonally by polymerase chain reaction, and protein16 with immunohistochemical method. In HPV positive cases, the presence of HPV types 16, 18 were evaluated by polymerase chain reaction. Demographic features of patients were noted. Patient survival and association with HPV/protein16 was determined.

**Results:**

Polyclonal HPV positivity was detected in 11 (12.2%) of 90 cases. Out of these 11 cases, HPV 16 was positive in 6, HPV 18 in 4, and both HPV 16 and 18 were positive in 1. In 18 (20%) of the cases, p16 was positive. Six of the cases (6.6%) had both HPV and protein16 positivity. In cases where protein16 alone or HPV and protein16 were co-positive, alcohol use was less and the tumor was found more likely to be localized in the supraglottic area. These ratios were statistically significant. Supraglottic localization of tumor was determined to be increased in protein16 positive cases. The correlation between protein16 positivity and supraglottic area location was determined to be statistically significant (*p* =  0.011). 55.6% of protein16 positive cases was located in the supraglottic region, 33.3% was glottic and 11.1% was transglottic. Although life expectancy over 5 years were numerically higher in HPV and protein16 positive cases, this was not found to be statistically significant. There was no statistically significant relationship between HPV positivity and mean age, differentiation, smoking and alcohol use, tumor progression, lymph node metastasis, localization, recurrence, cause of mortality and treatment methods in our study. The mean follow-up period of our patients was 6.7 years.

**Conclusion:**

The close relationship between HPV and oropharyngeal squamous cell carcinoma could not be shown in larynx malignancy in many studies, including our study. Our findings support a limited role of HPV in laryngeal carcinogenesis. Protein16 is not a reliable surrogate for HPV status in laryngeal cancers and is not a predictor of laryngeal cancer survival. Supraglottic localization of tumor was determined to be increased in protein16 positive cases. The correlation between protein16 positivity and supraglottic area location was determined to be statistically significant. There is a need for more populated clinical trials, where neoplastic proliferation is better demonstrated and the accuracy of the results obtained is supported by different techniques.

## Resumo

**Introdução:**

O papilomavírus humano é um fator de risco etiológico para um subconjunto de carcinoma espinocelular de cabeça e pescoço. Tem sido demonstrado que o HPV é um poderoso biomarcador prognóstico para o câncer de orofaringe, mas seu papel na laringe ainda não foi explorado em profundidade. Os mecanismos de desenvolvimento dos carcinomas de laringe são bastante complexos e controlados por vários fatores. Tabagismo e álcool são os fatores de risco mais importantes. Estudos recentes indicam que a infecção pelo HPV também desempenha um papel importante nos carcinomas da laringe. Os carcinomas laríngeos relacionados ao HPV ocorrem especialmente na região supraglótica.

**Objetivo:**

Nosso objetivo foi determinar a frequência da positividade para o HPV/proteína 16 em pacientes com carcinoma da laringe e a associação da positividade para o HPV e/ou proteína 16 com variáveis como idade, sexo, tabagismo, localização do tumor, metástase linfonodal, recidiva e sobrevivência de carcinoma da laringe em estágio avançado em nosso estudo.

**Método:**

Este estudo retrospectivo incluiu 90 pacientes com carcinoma laríngeo avançado. O grupo controle incluiu 10 amostras de mucosa laríngea normal. A presença de HPV foi investigada por anticorpo policlonal através de reação de polimerase em cadeia e a proteína 16 por método imunohistoquímico. Nos casos positivos para o HPV, a presença dos tipos 16 e 18 do foi avaliada por reação de polimerase em cadeia. As características demográficas dos pacientes foram observadas. A sobrevida dos pacientes e a associação com HPV/proteína 16 foram determinadas.

**Resultados:**

A positividade com anticorpo policlonal do HPV foi detectada em 11 (12,2%) dos 90 casos. Desses 11 casos, o HPV 16 foi positivo em 6, o HPV 18 em 4 e o HPV 16 e 18 foram positivos em 1. Em 18 (20%) dos casos, a proteína 16 foi positiva. Seis dos casos (6,6%) apresentaram positividade para HPV e proteína16. Nos casos positivos apenas para a proteína 16 ou quando HPV e a proteína 16 foram co-positivos, a ingestão de álcool foi menor e o tumor apresentou maior probabilidade de estar localizado na área supraglótica. Essas proporções foram estatisticamente significantes. A localização supraglótica do tumor foi maior em casos positivos para proteína 16. A correlação entre positividade para proteína 16 e localização da área supraglótica foi estatisticamente significante (*p* = 0,011). Dos casos positivos para proteína 16, 55,6% foram supraglóticos, 33,3% glóticos e 11,1% transglóticos. Embora a expectativa de vida acima de 5 anos tenha sido numericamente maior nos casos positivos para HPV e proteína 16, isso não foi estatisticamente significante. Não houve relação estatisticamente significante entre positividade do HPV e média de idade, diferenciação, tabagismo e uso de álcool, progressão tumoral, metástase linfonodal, localização, recidiva, causa de mortalidade e métodos de tratamento em nosso estudo. O período médio de seguimento de nossos pacientes foi de 6,7 anos.

**Conclusão:**

A estreita relação entre HPV e carcinoma espinocelular orofaríngeo não pôde ser demonstrada na laringe em muitos estudos, inclusive no nosso estudo. Nossos achados confirmam um papel limitado do HPV na carcinogênese da laringe. A proteína 16 não é um substituto confiável para o status do HPV nos cânceres de laringe e não é preditor da sobrevida do câncer de laringe. A localização supraglótica do tumor foi maior em casos positivos para proteína16. A correlação entre positividade para proteína 16 e localização na área supraglótica foi determinada como estatisticamente significante. Há necessidade de ensaios clínicos com amostras maiores, nos quais a proliferação neoplásica seja melhor demonstrada e a precisão dos resultados obtidos seja apoiada por diferentes técnicas.

## Introduction

Laryngeal cancers comprise more than 3 % of all malignant tumors in the body, and is the sixth most common cancer type around the world.^1^ Approximately 151,000 new diagnoses and 90,000 deaths have been reported annually due to laryngeal cancers.^2^ More than 95 % of laryngeal cancers are squamous cell carcinomas.^1^ The most significant factors in survival and recurrence of the disease are age, tumor stage and lymph node involvement, however, in recent years, certain etiological factors have shown importance in tumor development and prognosis.^3^ As in most head and neck cancers, the most important risk factors in the development of these cancers are smoking and alcohol abuse.^4^ It has been stated in recent studies that Human Papillomavirus (HPV) may also play a role in the etiology of Laryngeal Squamous Cell Carcinoma (LSCC).^5^, ^6^, ^7^, ^8^ HPV prevalence in laryngeal squamous cell carcinoma has been reported to vary between 20 % and 30 % in meta-analyses.^9^, ^10^ The most isolated HPV type in laryngeal cancers is HPV type 16, followed by HPV type 18.^9^, ^11^ P16 is an important suppressor gene in the control mechanism of cell cycle. Normally, p16 protein is present at very low levels in cells without dysplasia, and it cannot be determined with immuno-histochemical methods.^12^ Due to the transforming activity of E7 oncogen in all high-risk HPV types, p16 shows strong over-expression in dysplastic cervical cells and it can easily be shown by immuno-histochemical methods.^13^ High p16 expression and HPV positivity together show high correlation as infection indicators.^14^ The purpose of this study is to assess the effect of HPV type 16 and 18 infections and p16 expression on patients with late stage laryngeal cancer, and their prognostic contribution.

## Methods

Ninety patients diagnosed with advanced stage epidermoid (squamous cell) carcinoma of the larynx in Cukurova University School of Medicine Department of Otolaryngology between January 2006 ‒ December 2011, who were treatment-naive before, have been included in the study. This study has been approved by the local ethics committee of Cukurova University (Approval number: 20/48/2015). Cases with early stage laryngeal cancer, cases that have been diagnosed and treated before, and cases that cannot be followed up regularly have been excluded from the study.

### Immuno-histochemical assessment for p16

Paraffin-biopsy tissues fixed in formaldehyde were used with immuno-histochemical staining method for p16 and 10 normal larynx tissues were used for control group. Tissues detected in 10 % formaldehyde were blocked after tissue follow-up process and Hematoxylin-Eosin (HE) stained preparations were prepared in 5 μm serial cross-sections. They were examined by light microscope. Histological cross-sections were transferred on polylysine slides specific for immuno-histochemical staining. For PCR, 5–10 sheets of 5µ were sampled. For cases taken from the study group, p16 (Ventana, 760‒500) was applied to the cross-sections prepared from paraffin blocks with Streptavidin-Biotin complex immuno-peroxidase method. Normal larynx mucosa was used for all antibodies as positive control. Prepared samples have been assessed in light microscope.

### HPV typology

Firstly, polyclonal HPV, and then HPV 16 and 18 studies were performed on the cross-sections of cases prepared from paraffin blocks. General HPV 16 and 18 types were studied with PCR method.

### Statistical analysis

IBM SPSS Statistics Version 20.0 package program has been used in the statistical analysis of cases. Categorical measurements have been summarized in number and percentage, and numeric measurements as mean and standard deviation. Chi-square test statistics have been used in the comparison of categorical measurements between groups. Independent *t*-test has been used in the comparison of numeric measurements between groups. The McNemar test has been used for examining concordance between HPV and P16. Statistically significant level was assumed as 0.05 in all tests.^15^

## Results

Eighty-four (93.3 %) of the cases were male and 6 (6.7 %) were female. The average age was 59.9 + 10.8 (26–83). 46 cases were well-differentiated (51.1 %), 28 (31.1 %) were moderately-differentiated and 16 (17.8 %) were poorly differentiated. 44 tumors (48.9 %) had glottic, 24 (26.7 %) had supraglottic, 22 (24.4 %) had transglottic location. 60 cases (66.7 %) with T3 have been followed-up, and 30 cases (33.3 %) with T4 have been followed-up. Lymph node (LN) metastasis was determined in 29 (32.2 %) patients. While all patients included in the study had a history of smoking, 52 cases (57.8 %) were determined to have regular alcohol use. 27 cases (30 %) were determined to have recurrence, while no recurrence was determined in 63 (70 %) cases. Nine cases (10 %) underwent surgery only as primary treatment, while 40 (44.4 %) underwent post-operative adjuvant radiation, 28 (31.1 %) underwent post-operative adjuvant chemoradiotherapy and 13 (14.4 %) underwent only chemoradiotherapy without surgery. The average follow-up period was determined to be 6.7 years in our patients. Fifty-three (58.9 %) cases had a survival above 5 years, and 37 (41.1 %) had 5 year survival and below. Twenty one of 37 patients (56.8 %) died due to laryngeal cancer, and 16 (43.2 %) died due to others reasons such as cerebrovascular attack, coronary artery disesase and pneumonia.

Eleven cases (12.2 %) were determined to have polyclonal HPV positivity with PCR method. HPV 16 was positive in 6 of these 11 cases (54.5 %), HPV 18 was positive in 4 (36.4 %) and HPV 16 and 18 were positive in 1 case (9.1 %). p16, which was determined with immuno-histochemical staining method, was positive in 18 cases (20 %). 6 cases (6.6 %) had both HPV and p16 positivity. The relation between HPV and p16 test results was determined to have non-compliance.

In our study, supraglottic localization of tumor was determined to be increased in p16 positive cases. The correlation between p16 positivity and supraglottic area location was determined to be statistically significant (*p* =  0.011). 55.6 % of p16 positive cases was supraglottic, 33.3 % was glottic and 11.1 % was transglottic. In cases with any one of or both of HPV and p16 positivity, location of tumor in the supraglottic area was determined to be increased. The correlation between any one of or both HPV and p16 positivity, and supraglottic area location was determined to be statistically significant (*p* =  0.008). 52.2 % of these cases were supraglottic, 34.8 % was glottic and 13 % was transglottic.

Alcohol use was determined to be lower in p16 positive cases. The correlation between p16 positivity and alcohol use was determined to be statistically significant (*p* =  0.004). 72.2 % of p16-positive cases did not use alcohol and 27.8 % used alcohol.

Alcohol use was also determined to be lower in HPV and p16 positive cases. The correlation between HPV and p16 positivity and alcohol use has been determined to be statistically significant (*p* =  0.009). 64.7 % of HPV and p16 positive cases did not use alcohol, while 33.3 % did.

No statistically significant relation was determined between both HPV and p16 positivity and average age, tumor differentiation, smoking, stage, LN metastasis, recurrence, cause of death and treatment methods (Table 1).

**Table 1:** Relationship between HPV and p16 positivity and clinic parameters.

	Both positive	Only one positive	Both negative	*p*
Age	58.83 (12.281)	57.41 (10.063)	60.70 (10.909)	0.520
Differentiation				0.840
Well	2 (33.3 %)	8 (47.1 %)	36 (53.7 %)	
Moderate	2 (33.3 %)	6 (35.3 %)	20 (29.9 %)	
Poor	2 (33.3 %)	3 (17.6 %)	11 (16.4 %)	
Location				0.027
Glottic	3 (50.0 %)	5 (29.4 %)	36 (53.7 %)	
Supraglottic	2 (33.3 %)	10 (58.8 %)	12 (17.9 %)	
Transglottic	1 (16.6 %)	2 (11.8 %)	19 (28.4 %)	
Stage				0.468
T3	3 (50.0 %)	13 (76.5 %)	44 (65.7 %)	
T4	3 (50.0 %)	4 (23.5 %)	23 (34.3 %)	
Lymph node metastasis				0.589
Positive	3 (50.0 %)	6 (35.3 %)	20 (29.9 %)	
Negative	3 (50.0 %)	11 (64.7 %)	47 (70.1 %)	
Cigarette use (pack/year)	57.50 (23.187)	41.47 (14.975)	49.03 (17.061)	0.105
Alcohol use				0.009
Use	2 (33.3 %)	5 (29.4 %)	45 (67.2 %)	
Non-use	4 (64.7 %)	12 (70.6 %)	22 (22.8 %)	
Treatment				0.687
Surgery	0 (0.0 %)	3 (17.6 %)	6 (9.0 %)	
Surgery + Radiotherapy	2 (33.3 %)	8 (47.1 %)	30 (44.8 %)	
Surgery + Chemoradiotherapy	2 (33.3 %)	4 (23.5 %)	22 (32.8 %)	
Chemoradiotherapy	2 (33.3 %)	2 (11.8 %)	9 (13.4 %)	
Recurrence				0.291
** +**	3 (50.0 %)	3 (17.6 %)	21 (31.3 %)	
–	3 (50.0 %)	14 (82.4 %)	46 (68.7 %)	
Cause of death				0.128
Larynx ca	1 (33.3 %)	3 (100.0 %)	17 (54.8 %)	
Other	2 (66.7 %)	0 (0.0 %)	14 (45.2 %)	

**Figure 1:**
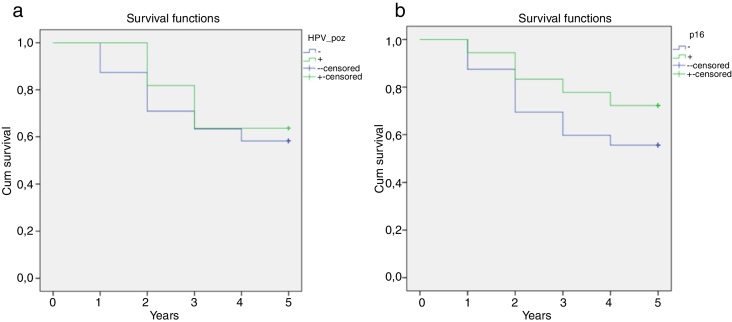
Correlation between HPV (a) and p16 (b) positivity and 5 year-survival.

Five-year survival of late stage LSCC cases in our study was determined to be 57.8 %. While 5 year survival of HPV-positive cases was determined to be 63.6 %, this rate was 57 % in HPV negative cases. Five-year survival of p16 positive cases was 72.2 %, while it was 54.2 % in p16-negative cases. While more than 5 years of survival was higher in HPV and/or p16-positive cases, this value was not determined to be statistically significant (Fig. 1).

## Discussion

Smoking and alcohol use are fundamental risk factors for the etiology of LSCC. HPV is a risk factor independent from smoking and alcohol intake. There are studies showing increased HPV incidence in cases that do not use cigarettes and alcohol.^16^, ^17^ The rate of HPV detection in LSCC cases is reported to be within the wide range of 0%–85% in reported studies.^8^, ^18^, ^19^ The reason for this wide range is the selection of patients, geographical differences, smoking and alcohol use, tumor location and differences in HPV diagnostic tests. In the meta-analysis performed by Li et al. HPV infection was determined in 28 % of cases, and a strong correlation was detected between HPV and LSCC.^19^ It has been shown in clinical studies that HPV plays a significant role particularly in LSCC pathogenesis of HPV-16.^20^ HPV positivity has been determined as 5 % in Germany, 4 % in France, and 23 % in USA.^20^ 11 (12.2 %) of 90 patients in our study was determined as HPV positive. Six of HPV positive cases (54.5 %) were determined to be p16-positive.

In a study performed by Ndiaye and colleagues, 649 of (22.1 %) 2739 LSCC cases were reported to have HPV positivity.^21^ No significant relation was determined between HPV prevalence and alcohol use, and gender in the same study. It was shown that HPV positivity was higher in people who did not use cigarettes or alcohol; however, it was not determined to be statistically significant. According to tumor location, 30 % of HPV positive cases was determined as supraglottic and 34 % as glottic. In a study, 33 of 674 LSCC cases (4.9 %) were determined to have HPV positivity.^16^ HPV positivity was determined to be statistically significantly higher in people who did not use cigarettes and alcohol, and people with masses in the supraglottic area. Similarly, it was determined in our study that HPV positivity was higher in people who did not use cigarettes and alcohol, and people with tumors in supraglottic area, in addition, HPV and p16 positivity was determined to be statistically significant with regard to alcohol use and tumor location (supraglottic location).

While HPV positive cases in the oropharynx area showed significantly better prognosis compared to HPV negative ones in most of the studies, this was not proven in Non-Oropharyngeal Head and Neck Cancers (NOSCC).^17^, ^22^ In the study performed by Duray et al., no significant difference was reported in LSCC HPV positive and negative cases with regard to smoking, age, stage, differentiation, recurrence and survival. Different from other studies, stage IV LSCC cases were shown to have higher HPV positivity compared to stage I and II cases in the same study.^23^ A similar study by Meshman et al. showed that p16 expression and presence of HPV in laryngeal and hypopharyngeal carcinomas have no correlation to overall survival and locoregional control.^24^ In our study, no statistically significant relation was determined between both HPV and p16 positivity and average age, differentiation, smoking, stage, LN metastasis, recurrence, cause of death and treatment methods.

Morshad et al. have performed one of the largest studies in HPV associated laryngeal cancers. In this study, no significant difference was determined with regard to survival between HPV positive and negative patients in their follow-up in year 3 and 5 after diagnosis.^25^ Average follow-up of the cases in our study have been determined as 6.7 years. Five-year survival rate was 57.8 %, and this rate was 63.6 % in HPV-positive cases and 57 % in HPV-negative cases.

p16 positivity rate in laryngeal cancers has been reported at rates varying from 1 % to 58 % in literature. In the study performed by Xu et al., 51 of 674 LSCC cases (7.57 %) were shown to have p16 positivity, while Chung et al. were determined to have p16 positivity in 11 of 140 LSCC cases (7.8 %) and also Kanyılmaz et al. have reported p16 positivity in 58 (44 %) of 131 LSCC cases.^16^, ^26^, ^27^ In our study, 18 of 90 patients (20 %) were determined as p16-positive.

There are studies showing cigarette and alcohol use is lower in p16 positive cases.^16^, ^17^, ^27^ In the study performed by Kalfert et al., 6 of non-smoking 8 cases was determined as p16-positive and assessed to be statistically significant.^28^ In the same study, it was stated that p16 might be a suitable immuno-histochemical indicator in non-smoking laryngeal cancer cases. In our study, 13 of 18 cases (72.2 %) that was determined to be p16-positive did not use alcohol. This result was determined to be statistically significant. All p16 positive patients were smokers.

There are various studies in the literature about p16 positivity and tumor location. Xu et al. reported that p16 positivity was significantly higher in supraglottic cases in their study.^16^ Although this relation was determined in other studies, a significant difference could not be shown.^27^, ^28^, ^29^ Ten (55.6 %) of 18 p16-positive cases in our study had supraglottic location and this value was determined to be statistically significant.

While some authors associated p16 presence in LSCC with good prognosis, p16 negativity was associated with poor prognosis and recurrence.^26^, ^27^, ^30^ Geisler and colleagues have reported in their study including 190 LSCC patients that p53 showed correlation with poor prognosis while p16 expression is not associated with prognosis.^31^ In our study, 5 year survival rate of p16 negative cases was 54.2 %, while 5 year survival rate of p16 positive cases was 72.2 %. Although the survival of p16 positive cases was better, it was not statistically significant.

In the study performed on the presence of recurrence by Kalfert et al., 8 of 58 LSCC cases (13.8 %) experienced recurrence 3 years after initial diagnosis. It was stated that all recurring cases had p16 negativity.^28^ In another study, while p16 positivity showed significantly good prognosis in oropharyngeal squamous cell carcinoma (OSCC), this significance was not shown in LSCC.^17^ In our study, 5 of p16-positive 18 cases (27.8 %) had recurrence in 5 years. The relation between p16-positivity and recurrence was not determined to be statistically significant.

While a relation between p16 positivity in OSCC, and low T stage and Lymph Node (LN) metastasis was shown, this relation could not be shown in LSCC cases.^32^ A significant relationship was determined between p16 and female gender and the presence of LN metastasis; no significant relation could be found with other clinical and demographic characteristics in the study of Young and colleagues.^29^ In our study, 8 of p16 positive 18 cases (44.4 %) were determined to have LN metastasis, but this result was not determined to be statistically significant. No relationship has also been determined between p16 positivity, and gender and tumor differentiation.

p16 positivity is an important indicator in the diagnosis of HPV infection. In more advanced and sensitive methods, different results on HPV-p16 correlation can be shown. In a study, HPV was determined to be positive in 33 of 674 LSCC cases, while p16 was positive in 51. All of HPV positive cases were also determined to be p16-positive, and p16 sensitivity was stated as 100 % and p16 specificity was stated as 65 %.^16^ These results are similar with the results of the study by Smeets et al. (p16 sensitivity 100 %, p16 specificity 79 %).^33^ In our results, p16 was determined to be positive in 6 of 11 HPV positive cases. p16 sensitivity was determined as 54.5 % and p16 specificity was 84.8 %. No statistically significant correlation was determined between p16 and HPV.

While it was shown that HPV and p16 positivity together provided better prognosis in OSCC, no significant result could be obtained in LSCC causes which may be due to the low number of HPV and p16 positive cases.^34^ Both p16 and HPV positivity show significantly better prognosis in NOSCC. In the study performed by Lewis et al., it was shown that there was no significant relation between HPV(-)/p16(+) cases and HPV(+)/p16(+) cases, these cases showed better prognosis compared to HPV(-)/p16(-) cases, and that p16 expression may be an indicator of good prognosis.^35^ We found that both HPV and p16 positive cases had lower recurrence and longer survival, but it was not determined to be statistically significant.

## Conclusion

Today, the accepted opinion for oncogenesis is that there should be a reciprocal interaction between different molecular factors for the formation of tumor. These factors are protooncogens, tumor suppressor genes and some viral oncogens that regulate cellular proliferation, apoptosis and differentiation. However, due to various factors such as cell type, cell environment and genetic background, it is hard to estimate the process of progress once carcinogenesis activity starts.

The close relationship between HPV and OSCC could not be shown on larynx in many studies including our study. Our findings support that p16 is not a reliable surrogate for HPV status in laryngeal cancers and is not a predictor of laryngeal cancer survival. Supraglottic localization of tumor was determined to be increased in p16 positive cases. The correlation between p16 positivity and supraglottic area location was determined to be statistically significant. Larger studies on HPV and p16 positivity in laryngeal cancer will be more useful for treatment, follow-up and prognosis of patients.

## Funding

Cukurova University Scientific Research Project Fund.

## Conflicts of interest

The authors declare no conflicts of interest.
